# Microbial-type terpene synthases enable enhanced insect and fungal resistance in engineered plants

**DOI:** 10.1016/j.bidere.2026.100087

**Published:** 2026-04-20

**Authors:** Saif ul Malook, Xinlu Chen, Bode A. Olukolu, Alessandro Occhialini, Scott C. Lenaghan, C. Neal Stewart, Feng Chen

**Affiliations:** a Center for Agricultural Synthetic Biology, University of Tennessee Institute of Agriculture, Knoxville, TN, 37996, USA; b Department of Plant Sciences, University of Tennessee, Knoxville, TN, 37996, USA; c Department of Entomology and Plant Pathology, University of Tennessee, Knoxville, TN, 37996, USA; d Department of Food Science, University of Tennessee, Knoxville, TN, 37996, USA

**Keywords:** Terpenes, Sesquiterpene, Metabolic engineering, Chemical defense, Insects

## Abstract

A major challenge in crop improvement is enhancing resistance to diverse biotic stresses. Because terpenoids play key roles in chemical defense, an envisioned strategy is to introduce new terpene metabolic pathways into crops through engineering. Microbial-type terpene synthase–like (*MTPSL*) genes are widespread in nonseed plants but absent in seed plants. Here, we engineered terpene metabolism in *Nicotiana benthamiana* using *MTPSL* genes, enabling production of sesquiterpenes absent in flowering plants and enhanced resistance to pest insects and fungal pathogens. Two liverwort *MTPSL* genes, *RlMTPSL3* and *RlMTPSL4*, which produce sesquiterpenes absent from flowering plants, were selected for metabolic engineering. In *N. benthamiana*, both genes generated sesquiterpenes consistent with their *in vitro* activities, and co-expression yielded combined profiles. Co-expression of *RlMTPSL3* and *RlMTPSL4*, individually or together, with 3-hydroxy-3-methylglutaryl-CoA reductase, the rate-limiting enzyme in sesquiterpene pathway, substantially increased sesquiterpene production. Bioassays of engineered tissues with two defoliating herbivores beet armyworm (*Spodoptera exigua*) and Colorado potato beetle (*Leptinotarsa decemlineata*) showed growth suppression and up to 30% mortality. The gut microbiome of beet armyworm feeding on engineered tissues showed differences from those feeding on control tissues, suggesting a potential mechanism underlying reduced pest insect performance. Engineered sesquiterpenes were recovered from larval frass, indicating stability through digestion. Transformed leaves emitted elevated sesquiterpenes as volatiles that repelled beet armyworm. In addition, extracts of engineered tissues inhibited the growth of *Fusarium oxysporum*, a fungal pathogen, by ∼50%. Together, these results demonstrate that *MTPSL*-based engineering can introduce new sesquiterpenes into flowering plants, providing a promising strategy for broad-spectrum crop protection.

## 1 Introduction

Plants, herbivorous insects, and microbial pathogens have coexisted for hundreds of millions of years, engaging in a continual evolutionary arms race in which they developed strategies to overcome or evade the defenses of the others [[1], [2], [3]]. This coevolution has driven the evolution of both direct defenses, such as physical barriers and toxic metabolites, and indirect defenses, such as traits that recruit natural enemies of herbivores or beneficial microbes that antagonize pathogens [4,5]. Chemical defenses are especially prominent: plants produce structurally diverse secondary metabolites that can deter feeding, inhibit growth, or attract predators, parasitoids, and microbial antagonists [[6], [7], [8]]. However, these metabolites are met with equally sophisticated detoxification and evasion mechanisms in insects and pathogens [9]. This ongoing coevolution presents a persistent challenge in agriculture, where pests rapidly adapt to plant resistance traits. One promising approach to address this challenge is to accelerate the evolution of secondary metabolism in crop plants through metabolic engineering, introducing biosynthetic pathways to produce new defense secondary metabolites.

One of the most important classes of plant secondary metabolites are terpenoids, which contribute to plant defense against insects through toxicity, feeding deterrence, and recruitment of beneficial organisms [10,11]. Terpenoids also play broad antimicrobial roles, functioning across plant lineages to inhibit pathogen growth and enhance immune responses [12]. Terpenoids are synthesized from the universal C_5_ building blocks isopentenyl diphosphate and dimethylallyl diphosphate, derived from the cytosolic/peroxisomal mevalonate (MVA) pathway or the plastidial methylerythritol phosphate (MEP) pathway [13]. Through the catalytic action of prenyltransferases, these precursors are sequentially condensed to form prenyl pyrophosphates including geranyl diphosphate (GPP, C_10_), farnesyl diphosphate (FPP, C_15_), and geranylgeranyl diphosphate (GGPP, C_20_). Terpene synthases then convert these substrates into a vast array of structurally diverse monoterpenes, sesquiterpenes, and diterpenes [[14], [15], [16]].

There are two types of terpene synthase genes in land plants, namely typical plant terpene synthase (*TPS*) genes or microbial terpene synthase-like (*MTPSL*) genes. TPSs are ubiquitous in land plants [17]. In contrast, *MTPSL* genes are restricted to non-seed plants [17]. Evolutionary studies suggest that *MTPSL* genes got lost in the common ancestor of seed plants [18]. In nonseed plants, MTPSLs are largely involved in making sesquiterpenes and monoterpenes [17,18]. There is also growing evidence supporting that MTPSL-formed terpenes function as defenses against herbivores and pathogens [19]. Some of their terpene products are absent in seed plants [18]. As such, the presence of *MTPSL* genes in nonseed plants and their absence in seed plants presents a unique opportunity for engineering seed plants for the production of new terpenes in seed plants using *MTPSL* genes. For this study, our initial objective was to determine whether *MTPSL* genes function effectively in flowering plants through metabolic engineering, using *Nicotiana benthamiana* as a model. Once validated, our next goal was to optimize MTPSL-based terpene pathways to enhance terpene production. Through accelerated pathway evolution, plants producing new terpenes may gain an advantage in the evolutionary arms race against insect pests and microbial pathogens. Finally, we assessed whether these newly introduced terpenes confer enhanced resistance to chewing insects and fungal pathogens, and investigated the possible mechanisms underlying enhanced insect resistance.

## 2 Materials and methods

### 2.1 Plant growth conditions

Seeds of *N. benthamiana* were sown in 10-cm pots containing a standard all-purpose peat-based growing mix (Berger BM2, https://www.berger.ca/en/) and maintained under controlled greenhouse conditions (22 ± 2 °C, 60–70% relative humidity, 16 h light/8 h dark photoperiod). Two-week-old seedlings were transferred to individual 10-cm pots. Plants were watered daily and fertilized weekly with nitrogen from Scout® Professional 15-16-17 Peat-Lite® Special Fertilizer (www.growwithpeters.com), applied as a drench. For agroinfiltration experiments, healthy and fully expanded leaves from 4-week-old plants were selected. Plants showing signs of mechanical damage, nutrient deficiency, or pest infestation were excluded.

### 2.2 Insect rearing and bioassay

Beet armyworm eggs were obtained from Benzon Research Inc., PA, USA (https://www.benzonresearch.com/). Eggs were incubated in a growth chamber at 27 °C, 60% relative humidity, and a 14 h light/10 h dark cycle until hatching (∼3 days). Freshly hatched neonates were used for mortality bioassays. The remaining larvae were reared on artificial diet (Southland *S. exigua* diet, Bio-Serv) in groups until second instar. Fresh agroinfiltrated leaf discs were placed on top of a solidified agar layer in each well to prevent leaf desiccation, and 1 s-instar larva was added per well for feeding assays. Insect rearing and all bioassays were conducted under the same controlled environmental conditions to ensure consistency in developmental rates and behavior. All equipment was sterilized between uses to minimize microbial interference in gut-based studies.

### 2.3 Volatile analysis by Gas Chromatography-Mass Spectrometry (GC/MS)

Agroinfiltrated leaf tissues were harvested for three days post-infiltration and flash-frozen in liquid nitrogen. Harvested tissues were ground with a mortar and pestle, and approximately 0.1 g of each sample was then suspended in 0.4 mL of 20% NaCl solution containing 0.001% 1-octanol as an internal standard. Volatiles were collected using solid-phase microextraction (SPME) fibers with a 100 μm polydimethylsiloxane (PDMS) (Supelco) at 50 °C for 30 min. For frass volatile analysis, frass samples were collected after 24 h of larval feeding and placed directly into GC/MS vials without solvent extraction. Volatile compounds were collected from the headspace using SPME fiber at 50 °C. For intact leaf headspace analysis, agroinfiltrated *N. benthamiana* leaves expressing *RlMTPSL3 + RlMTPSL4 + HMGR* were harvested, placed in sealed glass flasks, and volatiles were collected from the headspace using the same SPME conditions as described above. GC/MS analysis was performed on a Shimadzu GCMS-QP2010SE system equipped with an SH-I-5MS capillary column (30 m × 0.25 mm i.d., 0.25 μm film thickness). The injection port was set to 250 °C and operated in splitless mode. The oven program was as follows: initial temperature at 60 °C (held for 6 min), then increased at 5 °C/min to 300 °C. Helium was used as the carrier gas at a constant flow rate of 1 mL/min. Volatile compounds were identified by comparison of mass spectra with entries in the NIST 2020 and Wiley Registry 12th Edition libraries and confirmed by retention time matching with authentic standards where available. Kovats retention indices for terpenoids were calculated following the protocol described by Ref. [20]. C7 - C40 saturated alkanes standard (Sigma-Aldrich) was used for Kovats retention indention calculation.

### 2.4 Cloning and construct assembly

Previously *RlMTPSL3* (accession no. MZ367364) and *RlMTPSL4* (accession no. MZ367365) genes (Fan et al., 2021) were domesticated by removing *Bsa*I and *Bpi*I restriction sites and used to assemble level 0 modules with overhangs at 5′ and 3′ ends compatibles for Golden Gate cloning as described before [21,22]. Binary vectors for agrobacterium-mediated transformation of plant cells with *RlMTPSL3* and *RlMTPSL4* transgenes were assembled using Golden Gate modular cloning kits for plants as described before [21,22]. DNA construct sequences, including the promoter, coding sequence (CDS), and terminator are provided in Fig. S1. For this purpose, the MoClo Toolkit was a gift from Sylvestre Marillonnet (Addgene kit # 1000000044) [22,23], while the MoClo Plant Parts Kit was a gift from Nicola Patron (Addgene kit # 1000000047) [21]. Previously published level-0 modules [21], including the promoter-5′UTR from the Cassava Vein Mosaic Virus (CsVMV) (pICSL12006) and the *A. tumefaciens ocs* gene terminator (pICH41432) were used for *RlMTPSL3* and *RlMTPSL4* expression in plant tissue (Fig. S1). Constructs were verified by colony PCR, restriction enzyme digestion, and nanopore sequencing. All constructs were cloned using the *Escherichia coli* TOP 10 strain. Binary vectors for *HMGR* and *P19* expression were kindly provided by Dr. Tobias G Köllner as reported in Ref. [24] and introduced into *A. tumefaciens* strain LBA4404 for downstream applications.

### 2.5 *Agrobacterium*-mediated transient expression in tobacco

Binary vectors carrying *RlMTPSL3* or *RlMTPSL4* were transformed into chemically competent *Agrobacterium tumefaciens* LBA4404 using the freeze-thaw method as described previously [25]. Agroinfiltration experiments of *N. benthamiana* leaves were performed as previously described [26]. For this purpose, agrobacterium colonies carrying the construct of interest were selected on agar plates supplemented with rifampicin (50 μg/mL) and kanamycin (50 μg/mL) for plasmid selection and incubated at 28 °C for 2-3 days. A single colony was cultured overnight in 3 mL LB medium containing the same antibiotics at 28 °C with shaking at 200 rpm. Cells were harvested by centrifugation at 5,000×*g* for 5 min at room temperature, washed once with infiltration buffer (10 mM MES pH 5.6, 10 mM MgCl_2_, sterile water), and resuspended in infiltration buffer supplemented with 100 μM acetosyringone. The optical density at 600 nm (OD_600_) was adjusted to 0.5. Equal volumes of RlMTPSL constructs, HMGR and P19-containing cultures were mixed and incubated at room temperature for 2 h in the dark with gentle agitation.

Four-week-old *N. benthamiana* plants were watered the day before infiltration. Fully expanded young leaves were infiltrated on the abaxial side using a 1 mL needleless syringe. The suspension was gently pressed into the leaf until the solution spread visibly. Two to three infiltration spots were made per leaf, and the areas were marked with a permanent pen. Infiltrated plants were returned to the growth chamber and maintained under standard conditions (22 °C, 16 h light/8 h dark) for 3 days prior to sampling.

### 2.6 Insect feeding and mortality assays

Neonate larvae of beet armyworm were individually transferred onto freshly harvested agroinfiltrated *N. benthamiana* leaves collected 3 days post-agroinfiltration. A 16-well plate was used for both mortality and feeding assays. Each well was filled with 1 mL of 1% agar and allowed to solidify, after which a leaf disc was placed on the agar surface and a single larva was added. Two treatments were evaluated: (i)empty vector +*HMGR*+*P19;* and (ii) *RlMTPSL3*+*RlMTPSL4*+*HMGR*+*P19*. For both mortality and growth assays with BAW, five independent plants were used per treatment. Each plant was grown in an individual pot and considered an independent biological replicate. Four leaf discs were collected from each plant, resulting in a total of 20 discs (and 20 larvae) per treatment. Leaf discs were randomly assigned to wells to ensure independence among biological replicates. Larval mortality was recorded after 24 h of feeding; larvae were considered dead if they showed no movement upon gentle probing. For growth assays, larvae were allowed to feed for 48 h, after which individual body mass was measured using a precision balance (±0.1 mg). For Colorado potato beetle assays, five independent plants were used per treatment, from which a total of 16 leaf discs were collected. One first-instar larva was assigned per leaf disc (n = 16 per treatment) and allowed to feed for 48 h before weighing. All assays were independently repeated three times and performed in a controlled-environment chamber (27 °C, 60% RH, 14:10 h light:dark).

### 2.7 Insect behavioral assays

A two-choice behavioral assay was used to quantify larval preference between engineered and control *N. benthamiana* leaves harvested 3 days post-agroinfiltration. Third-instar larvae were starved for 2 h prior to testing and then introduced into a rectangular plastic arena (60 × 30 cm) divided into three zones: a central neutral release zone (20 cm) and two lateral choice zones containing leaf discs infiltrated with either the *RlMTPSL3 + RlMTPSL4 + HMGR + P19* construct (Engineered) or the *empty vector + HMGR + P19* control (Control). Leaf discs were collected from independently agroinfiltrated plants, with ten plants per treatment (one plant per pot) and one disc excised per plant, resulting in 10 discs per treatment. For each trial, ten engineered leaf discs (*RlMTPSL3* + *RlMTPSL4* + *HMGR* + *P19*) were placed in one lateral zone, and ten control leaf discs (empty vector + *HMGR* + *P19*) were placed in the opposite lateral zone. The positions of engineered and control treatments were alternated between trials to minimize positional bias. At the start of each assay, twenty larvae (*n* = 20) were placed in the central zone and allowed to disperse freely under constant light. After 20 min, the number of larvae present on the engineered leaf disc, on the control leaf disc, or remaining in the neutral zone (“No choice”) was recorded.

Nine trials (T1-T9) were conducted under identical conditions. Because these trials represent technical replicates, they were aggregated into three biological replicate groups (Group 1: T7-T9; Group 2: T4-T6; Group 3: T1-T3). Grouping prevented pseudoreplication and avoided inflated degrees of freedom, ensuring that statistical inference reflected biological rather than technical variation. This approach is consistent with established practice in behavioral and chemical-ecology experiments where repeated technical assays are averaged to generate a single biological datum.

For each trial, the proportion of larvae choosing each option was calculated as the number selecting that option divided by the total larvae present. Mean proportions for Engineered, Control, and No-choice outcomes were then derived for each biological group. To test the hypothesis that engineered leaves repel larvae, a one-tailed binomial test was performed for each group using raw counts of larvae selecting Engineered versus Control, with the null hypothesis of equal probability (*p* = 0.5) and the alternative hypothesis p (Engineered) < p (Control) [27]. Larvae remaining in the neutral zone were excluded from inferential testing following standard practice for two-choice assays.

### 2.8 Insect gut dissection and DNA extraction

Third-instar *S. exigua* larvae were fed for 48 h on detached *N. benthamiana* leaves infiltrated with either the *RlMTPSL3 + RlMTPSL4 + HMGR* construct or the empty-vector control. After feeding, larvae were surface-sterilized in 70 % ethanol for 1 min and rinsed three times with sterile phosphate-buffered saline (PBS, pH 7.4). Entire insect guts were dissected aseptically under a laminar-flow hood using flame-sterilized fine scissors and forceps. For each treatment, five independent guts were pooled as one biological replicate to minimize inter-individual variability. Samples were immediately frozen in liquid nitrogen and stored at −80 °C until extraction.

Total genomic DNA from dissected beet armyworm guts was extracted using the Aidlab CTAB Plant Genomic DNA Rapid Extraction Kit (DN14) (Aidlab Biotechnologies Co., Ltd., Beijing, China) following the manufacturer's protocol with minor modifications for animal tissue. Briefly, dissected midguts were homogenized in preheated lysis buffer PL (65 °C) containing 2% β-mercaptoethanol and incubated for 30 min to ensure complete cell lysis. After chloroform extraction, the aqueous phase was transferred to a new tube, mixed with binding buffer PQ (with ethanol added as recommended), and passed through the silica-based spin column for DNA adsorption. Bound DNA was washed twice with wash buffer WB, eluted in 50–100 μl of preheated elution buffer EB, and stored at −20 °C. DNA purity and concentration were assessed using a NanoDrop 2000 spectrophotometer (Thermo Fisher Scientific) and Quant-iT™ PicoGreen™ dsDNA Assay Kit (Invitrogen). The resulting DNA exhibited A260/A280 ratios of 1.7-1.9 and was used directly for downstream OmeSeq-qRRS library preparation and sequencing.

### 2.9 OmeSeq-qRRS library preparation, sequencing, and metagenomic profiling

OmeSeq-qRRS library preparation and sequencing were performed following established protocols [[28], [29], [30]]. Briefly, genomic DNA was quantified using the Quant-iT PicoGreen dsDNA Assay Kit (Thermo Fisher Scientific) and normalized to 20 ng μl^−1^ (minimum 1 ng μl^−1^). Sequential double digestion was carried out with NsiI-HF and NlaIII (New England Biolabs) while integrating custom Illumina P5 and P7 adapters containing 96 × 96 variable-length (7-10 bp) barcodes and unique molecular identifiers. The double-stranded DNA and incubation at 37 °C ensures that adapter annealing was restricted to the overhangs to prevent off-target binding. The single-stranded adapters were incorporated into genomic fragments through isothermal amplification using Bst 2.0 WarmStart DNA Polymerase (New England Biolabs). After first digestion and isothermal amplification, fragments less than 300 bp were excluded after Ampure XP bead (Beckman Coulter) purification. Equimolar amounts of samples were pooled after the sequential digest and isothermal amplification.

Fragments between 300 and 800 bp were obtained by double size selection using AMPure XP bead. Libraries were then amplified for 18 cycles of quantitative PCR and re-purified by an additional double size-selection step. The library was sequenced to generate 150 bp paired end read on a lane of theIllumina NovaSeq X Plus flow cell at Admera Health, New Jersey.

### 2.10 Demultiplexing and quality filtering

Raw reads were processed with ngsComposer to demultiplex and quality filter reads [28]. Quality control included trimming the 6-bp buffer sequence preceding each barcode, demultiplexing with ≤1 bp mismatch, retaining only reads with intact restriction motifs, and applying sliding-window trimming (10 bp window; Q ≥ 20). Reads shorter than 64 bp after trimming or with <80 % bases at Phred ≥20 were discarded. Adapter sequences were removed when ≥12 bp of overlap was detected. High-quality reads were exported for downstream metagenomic profiling.

### 2.11 Metagenomic profiling and taxonomic classification

Filtered reads were processed using the Qmatey pipeline (https://github.com/bodeolukolu/Qmatey) [30], which applies exact-matching algorithms for quantitative taxonomic profiling across all taxonomic groups on the NCBI database. Host-derived reads were removed via alignment to the *N. benthamiana* genome using BWA-MEM. Remaining reads were compressed, indexed, and aligned to the NCBI nt database using MEGABLAST. Exact-matching of consensus sequences enabled strain-to phylum-level classification. Resulting abundance tables were normalized by relative-sum scaling to obtain quantitative species-level profiles, which were used for downstream analyses.

### 2.12 Pathogen growth inhibition assay

The antifungal activity of terpenes produced by *RlMTPSL3* and *RlMTPSL4* was evaluated against *Fusarium oxysporum* f. sp. *lycopersici*. Four-week-old *N. benthamiana* plants were agroinfiltrated with *RlMTPSL3*+*RlMTPSL4*+*HMGR*+*P19*, or with an empty vector +*HMGR*+*P19* as a control. Three days post-infiltration, infiltrated leaves were harvested. Terpenoids and other insoluble metabolites were extracted by immersing leaf disks in ethyl acetate (0.4 mL solvent per 0.1 g tissue) for 3 h at room temperature with gentle shaking. Extracts were clarified by centrifugation (10,000×*g*, 10 min). Potato dextrose agar plates (9 cm diameter) were evenly coated with 400 μl of each extract using a sterile spreader and allowed air to dry under sterile conditions. A 5 mm diameter mycelial plug, excised from the actively growing margin of a 7-day-old *F. oxysporum* f. sp. *lycopersici* culture, was placed at the center of each plate. Plates were incubated at 28 °C in the dark for 7 days. Colony diameters were measured along two perpendicular axes and averaged for each plate. Three biological replicates (independent plant extractions) were used per treatment, with three technical replicates per extract.

### 2.13 Statistical analysis

Larval biomass, mortality, and fungal growth were analyzed using an unpaired two-tailed Student's *t*-test, with statistical significance set at *P* < 0.05. Data visualization and analysis were performed in R (v4.2.1) using the ggplot2 and agricolae packages [31,32]. Normalized abundance matrices were imported into R (v4.3.2) for diversity and compositional analyses. Alpha-diversity metrics (Richness) and beta-diversity (Bray-Curtis) were computed using the vegan package. Community differences were evaluated via Wilcoxon rank-sum tests and PERMANOVA (*adonis2*, 999 permutations). Visualization of taxonomic composition and ordination plots was performed with ggplot2 and ggpubr [33].

## 3 Results

### 3.1 Metabolic engineering using *RlMTPSL3* and *RlMTPSL4* in *N*. *benthamiana*

Among a growing list of *MTPSL* genes that have been functionally characterized are *MTPSL* genes isolated from the leafy liverwort *Radula lindenbergiana* [34]. Two of them, *RlMTPSL3* and *RlMTPSL4*, produce novel sesquiterpenes. RlMTPSL3 catalyzes the formation of five sesquiterpenes including along with additional products including pentalenene, asterisca-1,6-diene, asterisca-2 (9),6-diene, (E)-β-caryophyllene and α-humulene with asterisca-1,6-diene being the major product [35]. Asterisca-1,6-diene was known to be produced by some bacteria but has not been reported from any plant [35]. RlMTPSL4 is also a multi-product sesquiterpene synthase. It catalyzes the formation of six sesquiterpenes including β-elemene, 4,5-diepi-isoishwarane, 4,5-diepi-aristolochene, eremophilene, α-selinene and germacrene A with 4,5-diepi-isoishwarane being the major product [36]. 4,5-diepi-isoishwarane was a novel sesquiterpene and has not been reported from any organisms including plants [36]. Because of novel enzyme activity of RlMTPSL3 and RlMTPSL4, their encoding genes can be used for sesquiterpene engineering in flowering plants to produce new sesquiterpenes.

**Fig. 1:**
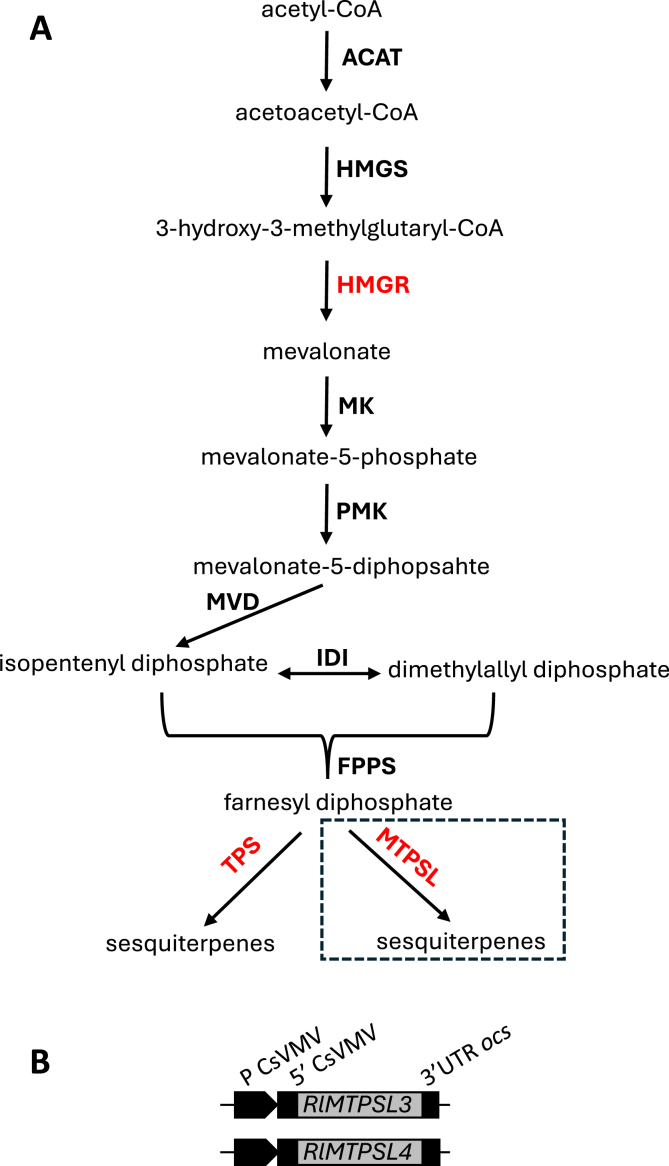
**Design of new sesquiterpene biosynthesis in seed plants.** (A), the general pathway leading to sesquiterpene biosynthesis in plants. The participating enzymes include acetyl-CoA C-acetyltransferase (ACAT), 3-hydroxy-3-methylglutaryl CoA synthase (HMGS), 3-hydroxy-3-methylglutaryl-CoA reductase (HMGR), mevalonate kinase (MK), phosphomevalonate kinase (PMK), mevalonate diphosphate decarboxylase (MVD), isopentenyl diphosphate isomerase (IDI), farnesyl diphosphate synthase (FPPS), typical plant terpene synthase (TPS) and microbial type terpene synthase (MTPSL). The boxed pathway naturally occurs only in nonseed plants. HMGR is the rate-limiting enzyme. TPS and MTPSL determine sesquiterpene product specificity. (B), Schematic representation of *RlMTPSL3* and *RlMTPSL4* expression constructs. The coding sequences of *RlMTPSL3* and *RlMTPSL4* were cloned downstream of the cassava vein mosaic virus promoter (CsVMV), including the 5′ CsVMV untranslated region (5′ UTR), and fused to the octopine synthase terminator (3′ UTR ocs). See Fig. S1 for the sequences of CsVMV, 3′UTR ocs, *RlMTPSL3* and *RlMTPSL4*.

**Fig. 2:**
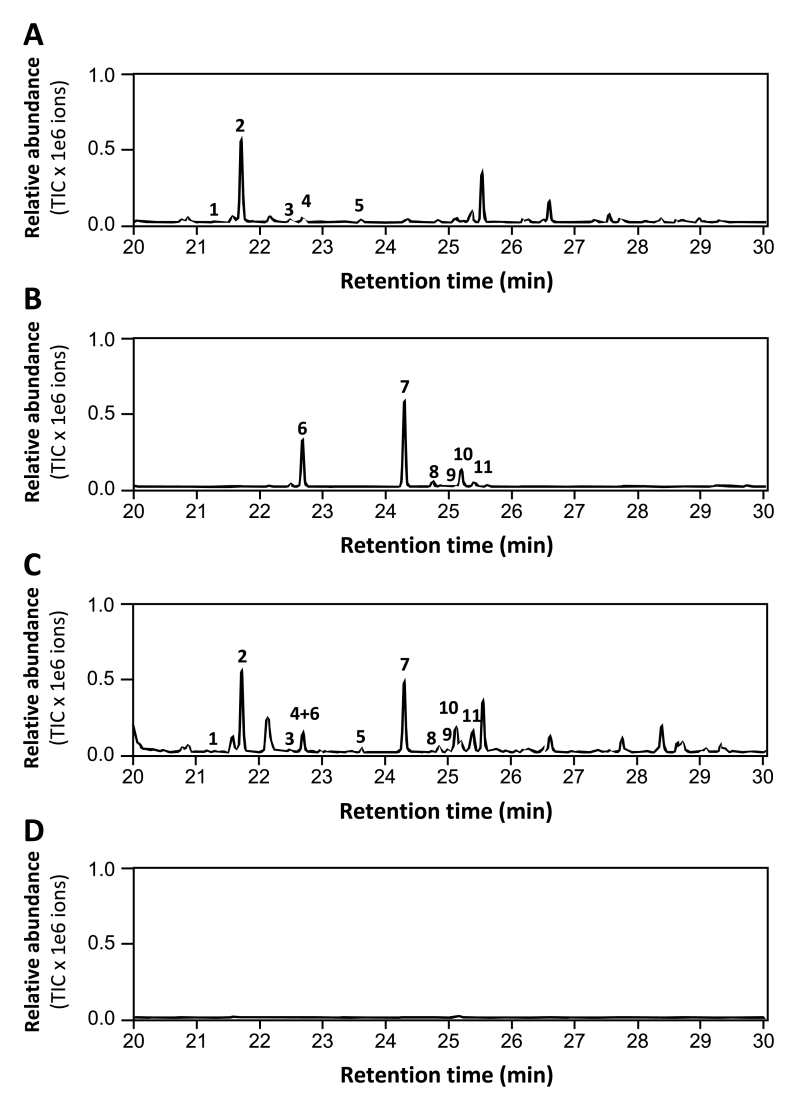
**GC/MS analysis of sesquiterpene production in *N*. *benthamiana* leaves after agroinfiltration.** Four-week-old plants were agroinfiltrated and sampled 3 days post-infiltration (dpi) for GC/MS analysis. (A) *RlMTPSL3* expression led to the production of five sesquiterpenes including pentalenene (peak 1), asterisca-1,6-diene (peak 2), asterisca-2 (9),6-diene (peak 3), (E)-β-caryophyllene (peak 4), and α-humulene (peak 5). (B) *RlMTPSL4* expression led to the production of six sesquiterpenes, including β-elemene (peak 6), 4,5-diepi-isoishwarane (peak 7), 4,5-diepi-aristolochene (peak 8), eremophilene (peak 9), α-selinene (peak 10), and germacrene A (peak 11) (C) *RlMTPSL3 + RlMTPSL4* co-expression yielded a combined profile containing the products of both RlMTPSL3 (peaks 1 to 5) and RlMTPSL4 (peaks 6 to 11). (D) Empty vector: no sesquiterpene peaks above background.

The natural sesquiterpene biosynthetic pathway can be divided into three stages (Fig. 1A): the production of two five carbon building isopentenyl diphosphate and dimethylallyl diphosphate through the MVA pathway, in which 3-hydroxy-3-methylglutaryl-CoA reductase (HMGR) is the rate-limiting enzyme [37], the production of farnesyl diphosphate, which is the substrate of sesquiterpenes and the production of sesquiterpenes from farnesyl diphosphate through the action of TPSs or MTPSLs. To determine whether the liverwort-derived *MTPSL* genes can function in flowering plants, *RlMTPSL3* and *RlMTPSL4* were constructed in binary vector (Fig. 1B) and transiently expressed in *N*. *benthamiana* leaves. This heterologous system allows direct evaluation of terpene biosynthetic activity and subcellular compatibility of ancient MTPSL enzymes within a modern plant host. Leaves infiltrated with *RlMTPSL3* and *RlMTPSL4* alone or in combination led to the production of new sesquiterpenes not produced by the wild type plants. Expression of *RlMTPSL3* led to the accumulation of major product, asterisca-1,6-diene along with additional products including pentalenene, asterisca-2 (9),6-diene, (E)-β-caryophyllene, and α-humulene (Fig. 2A), whereas that of *RlMTPSL4* led to the production of six sesquiterpenes, β-elemene, 4,5-diepi-isoishwarane, 4,5-diepi-aristolochene, eremophilene, α-selinene and germacrene A with 4,5-diepi-isoishwarane being the major product (Fig. 2B). Co-expression of *RlMTPSL3*, and *RlMTPSL4* produced the sesquiterpene products of both genes (Fig. 2C). No sesquiterpene products were detected in empty-vector controls (Fig. 2D).

### 3.2 Co-expression with HMGR enhanced sesquiterpene production

**Fig. 3:**
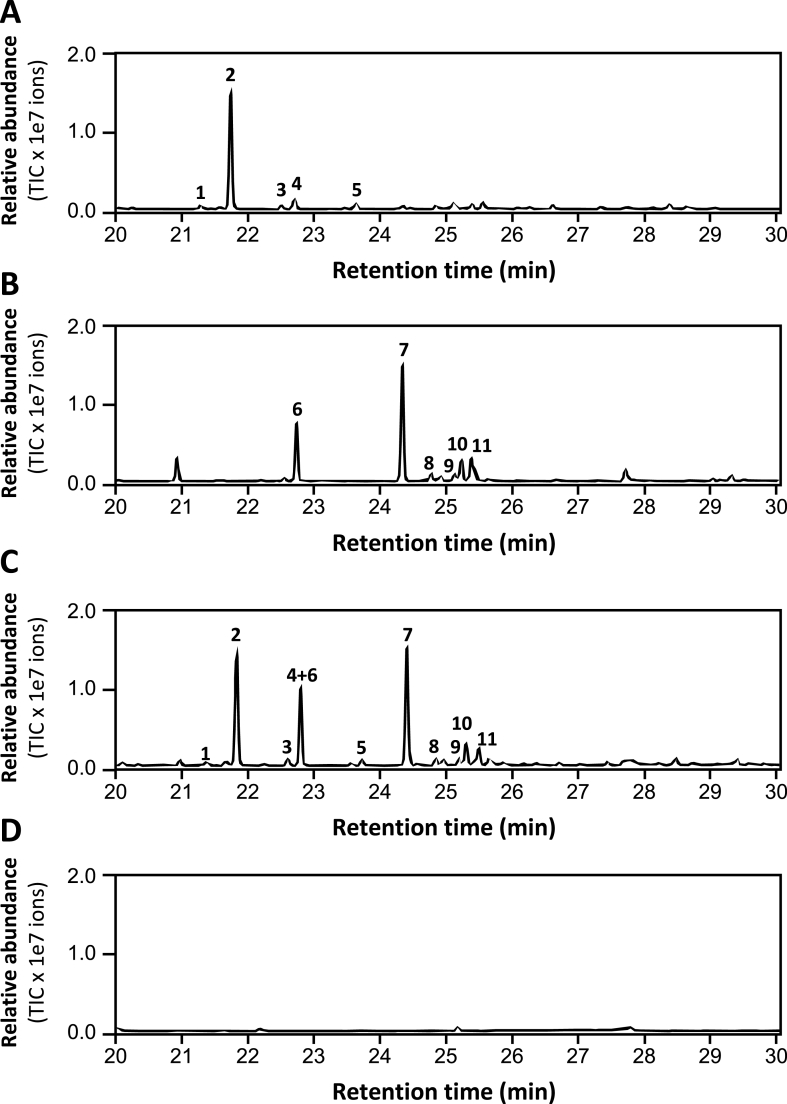
**GC/MS analysis of sesquiterpene production in agroinfiltrated *N*. *benthamiana* leaves with HMGR co-expression.** Four-week-old plants were agroinfiltrated and sampled 3 days post-infiltration (dpi) for GC/MS. (A) *RlMTPSL3 + HMGR* produced expression led to the production of five sesquiterpenes including pentalenene (peak 1), asterisca-1,6-diene (peak 2), asterisca-2 (9),6-diene (peak 3), (E)-β-caryophyllene (peak 4), and α-humulene (peak 5). (B) *RlMTPSL4*+*HMGR* expression led to the production of six sesquiterpenes, including β-elemene (peak 6), 4,5-diepi-isoishwarane (peak 7), 4,5-diepi-aristolochene (peak 8), eremophilene (peak 9), α-selinene (peak 10), and germacrene A (peak 11). (C) *RlMTPSL3 + RlMTPSL4 + HMGR* yielded a combined sesquiterpene profile containing products of both RlMTPSL3 (peaks 1 to 5) and RlMTPSL4 (peaks 6 to 11). (D) *Empty vector*+*HMGR*: no sesquiterpene peaks above background.

Based on the data that transit expression of *RlMTPSL3*, *RlMTPSL4* or *RlMTPSL3&4* led to the production of their respective *in vitro* sesquiterpene products (Fig. 3), next we asked the question whether increasing flux of the sesquiterpene biosynthetic pathway would enhance sesquiterpene yields. To this end, the respective genes were co-expressed with *HMGR*, the rate-limiting gene of the MVA pathway (Fig. 1). GC/MS analysis revealed that boosting the mevalonate pathway markedly enhanced sesquiterpene production compared to the leaves without *HMGR* (Fig. 3). In *RlMTPSL3*-expressing tissues, *HMGR* co-expression resulted in approximately a 34-fold increase in asterisca-1,6-diene relative to *RlMTPSL3* alone (Fig. 3A). For *RlMTPSL4*, *HMGR* co-expression produced roughly a 49-fold increase in β-elemene and a 72-fold increase in 4,5-diepi-isoishwarane compared with *RlMTPSL4* alone (Fig. 3B). When both *RlMTPSL3* and *RlMTPSL4* were co-infiltrated with *HMGR*, both sets of their respective sesquiterpene products were elevated (Fig. 3C). Similarly, no sesquiterpene was detected in plant tissues transformed with the empty vector (Fig. 3D).

### 3.3 *RlMTPSL3&4*-derived sesquiterpenes suppress growth of insects

**Fig. 4:**
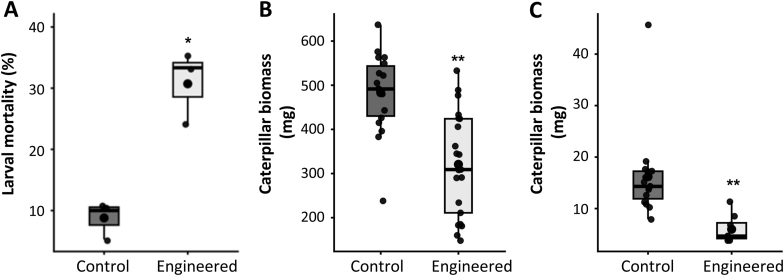
**Insect bioassay of beet armyworm on agroinfiltrated *N*. *benthamiana* leaves.** Four-week-old *N. benthamiana* leaves were agroinfiltrated with *RIMTSL3 + RIMTSL4 + HMGR* or an *empty vector + HMGR* as a control. Three days of post-infiltration, infiltrated leaf disks were excised and placed on agar in plastic containers. A) Neonates larvae were fed on agroinfiltrated leaves for 48 h and mortality data was recorded. B) Second instar beet armyworm larvae were introduced, and larval weight gain was recorded after 48 h of feeding. C) First instar Colorado potato beetle larvae were allowed to feed and weight gain was recorded 4 days of feeding. Data was analyzed by Student's *t-test* (two-sided, *P* < 0.05); asterisk indicates a significant difference between treatments.

Many terpenes function as direct chemical defenses against insects in plants by inhibiting insect growth [10,38,39]. We evaluated whether *RlMTPSL3&4*-derived sesquiterpenes impair herbivores using a stepwise design that first measured lethality and then sublethal growth. In a neonate assay with beet armyworm (*Spodoptera exigua*), larvae feeding on *RlMTPSL3&4*&*HMGR*-expressing agroinfiltrated *N. benthamiana* leaves showed substantially higher mortality than on the empty vector control (mean ± SE: 30.7 ± 6.0% vs 8.79 ± 3.11%, *n* = 16 per group; *p* = 0.011), a ∼3.5-fold increase (Fig. 4A). To assess growth effects in later instars, we performed feeding bioassays using agroinfiltrated *N. benthamiana* leaves. Beet armyworm second-instar larvae feeding on leaves expressing *RlMTPSL3&4*&*HMGR* exhibited a marked reduction in biomass. Mean larval mass decreased from 483.1 ± 22.1 mg in the empty-vector control to 320.8 ± 26.3 mg in the *RlMTPSL3*+*RlMTPSL4*+*HMGR* treatment (*n* = 20), corresponding to a 33.6% reduction in biomass (Fig. 4B). A similar trend was observed in the Colorado potato beetle (*Leptinotarsa decemlineata*) where larvae feeding on *RlMTPSL3&4*-expressing leaves showed the 71% reduction in biomass (Fig. 4C).

### 3.4 *RlMTPSL3/4*-derived terpenes reshape gut microbial communities in gut of beet armyworm

**Fig. 5:**
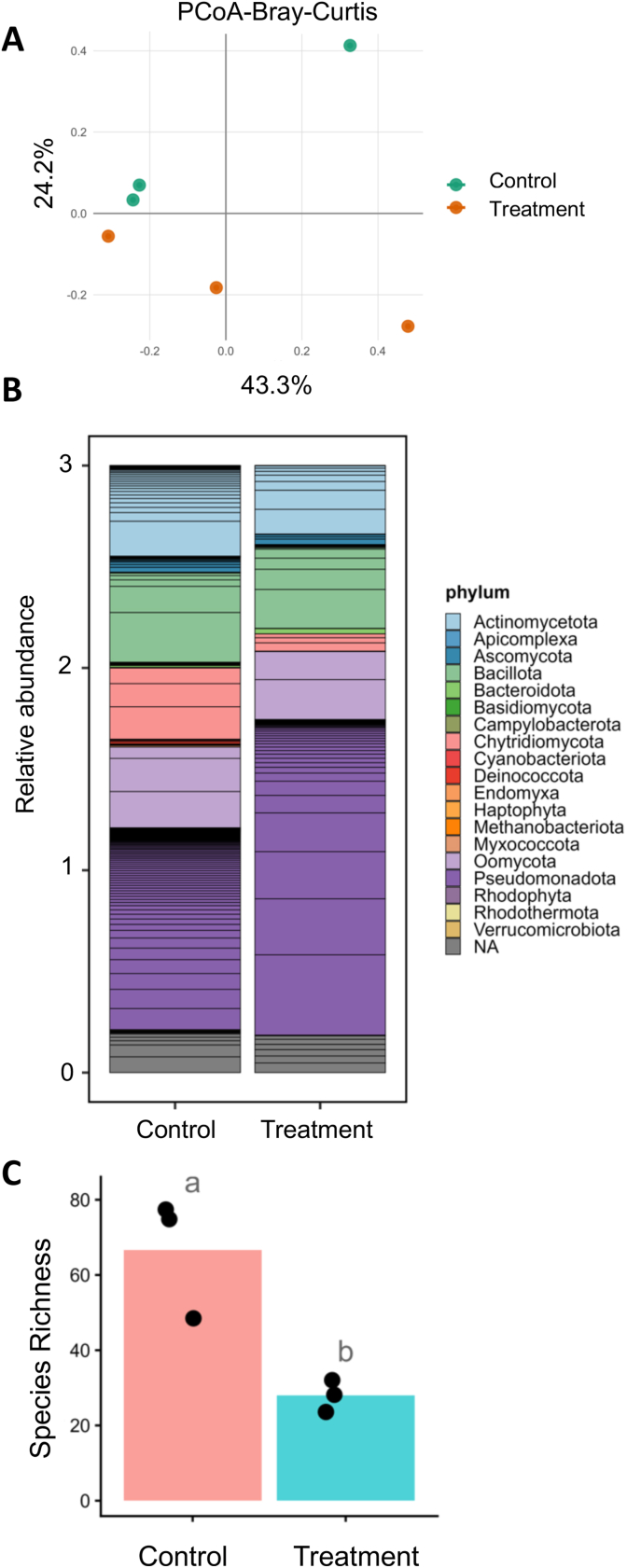
**Gut microbiome community structure and diversity in beet armyworm larvae fed on *N*. *benthamiana* leaves infiltrated with *RlMTPSL3*+*RlMTPSL4*+*HMGR*.** Four-week-old *N. benthamiana* plants were agroinfiltrated and harvested at 3 days post-infiltration (dpi). Third-instar beet armyworm (*S. exigua*) larvae were fed detached leaves for 48 h. Following feeding, larval guts were dissected and subjected to metagenome sequencing using the OmeSeq-qRRS platform. (A) Principal coordinates analysis (PCoA) is based on Bray–Curtis dissimilarities (PERMANOVA, R^2^ = 0.1, *p* = 0.05). (B) Relative abundance of dominant microbial taxa at the phylum level in larval gut samples from each treatment with stacked bars representing individual biological replicates. (C) Species richness of gut microbiota in insects fed on *RlMTPSL3/4*+ *HMGR*-infiltrated versus empty-vector (EV) leaves. Different letters indicate significant differences between treatments (*p* < 0.05).

Because ingested plant metabolites can remodel insect gut microbiota and thereby impose evolutionary pressure on herbivore detoxification and performance, we examined whether *RlMTPSL3*+*RlMTPSL4 +HMGR*-derived sesquiterpenes alter gut microbial structure in beet armyworm larvae. Larvae were fed on *N. benthamiana* leaves expressing *RlMTPSL3*+*RlMTPSL4*+*HMGR* and their gut microbiomes were profiled using OmeSeq. Bray-Curtis–based principal coordinates analysis (PCoA) revealed partial separation of gut microbial communities between larvae fed *RlMTPSL3*+*RlMTPSL4*+*HMGR* infiltrated leaves and those fed empty vector controls (Fig. 5A). PERMANOVA analysis indicated a marginal but detectable treatment effect on community structure (R^2^ = 0.198, *P* = 0.05), suggesting modest shifts in overall microbial composition associated with terpene ingestion. Consistent with these structural changes, taxonomic profiling revealed differences in the relative abundance of dominant microbial phyla between treatment groups (Fig. 5B). While both groups were dominated by taxa commonly reported in lepidopteran guts, larvae fed *RlMTPSL3*+*RlMTPSL4*+*HMGR*-expressing leaves exhibited reduced relative representation of fungus *Chytridiomycota*, whereas bacteria *Pseudomonadota* were more abundant in empty vector leaves fed larvae. In contrast to the moderate effects on beta diversity and composition, species richness differed strongly between treatments (Fig. 5C). Larvae fed *RlMTPSL3*+*RlMTPSL4*+*HMGR*-expressing leaves showed a significant reduction in gut microbial richness relative to empty vector-fed controls (*P* < 0.05), indicating a loss of detectable taxa following consumption of terpene-producing plant tissue.

### 3.5 Ingested *RlMTPSL3/4*-derived sesquiterpenes persist in frass

**Fig. 6:**
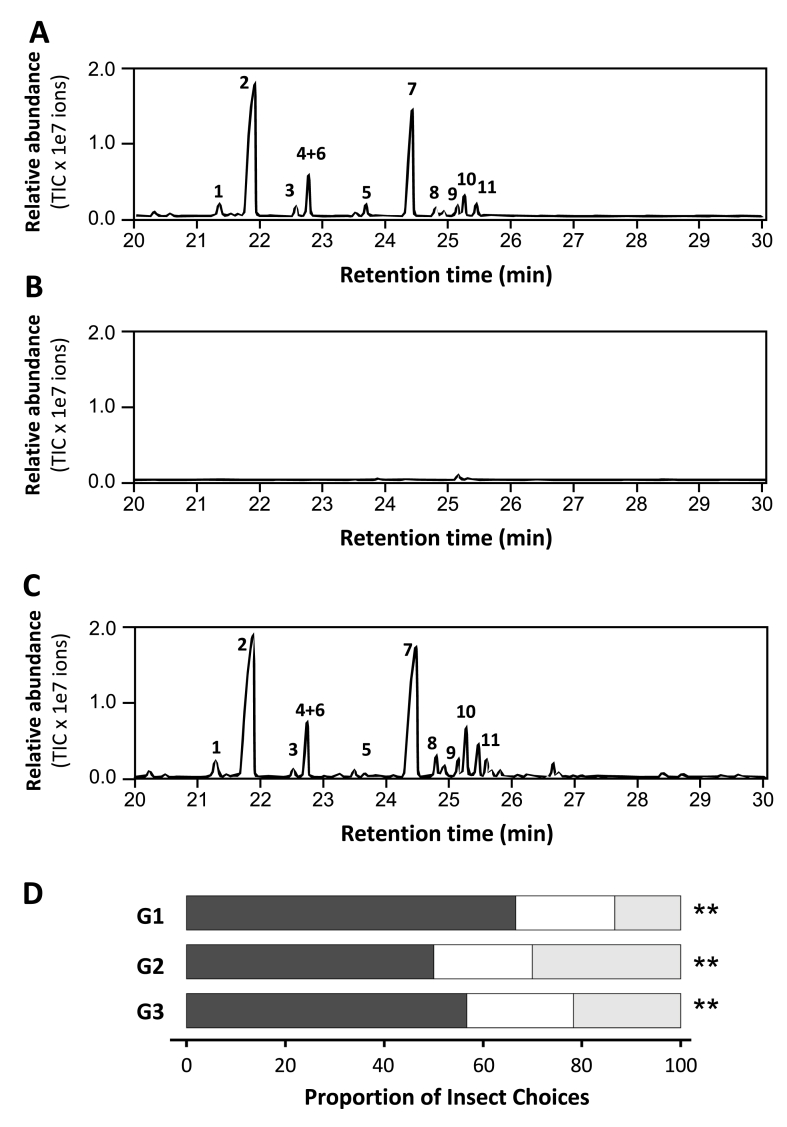
**Volatiles analysis and insect behavior.** (A) The frass of larvae fed on *RlMTPSL3 + RlMTPSL4 + HMGR* agroinfiltrated leaves yielded a combined sesquiterpene profile containing products derived from both enzymes, Four-week-old plants were agroinfiltrated and sampled at 3 dpi; third-instar beet armyworm were fed detached leaves for 48 h, and frass was analyzed by GC/MS. (B) *Empty vector + HMGR*: no sesquiterpene peaks were detected above background. (C) Headspace SPME-GC/MS analysis of intact leaves expressing *RlMTPSL3 + RlMTPSL4 + HMGR*. Four-week-old plants were agroinfiltrated and sampled at 3 days post-infiltration (dpi). (D) Two-choice behavioral assay used to quantify larval responses to engineered (*RlMTPSL3+ RlMTPSL4 + HMGR*) and control (*empty vector + HMGR*) leaves. Third-instar larvae were released into the central neutral zone of a three-section arena and allowed to disperse freely for 20 min. Black bars represent the mean proportion of larvae selecting the control leaf disc, the engineered leaf disc (light grey), or remaining in the no-choice zone (white), averaged across three biological replicate groups G1-G3 (each group consisting of three technical trials). Statistical comparison of engineered versus control choice was performed using a one-tailed binomial test with the null hypothesis of equal choice (*P* = 0.5) and the alternative hypothesis that larvae chose the engineered leaf less frequently (*P* < 0.5). Asterisks indicate significance levels (*P* < 0.05, P < 0.01, *P* < 0.001). The sesquiterpenes were pentalenene (peak 1), asterisca-1,6-diene (peak 2), asterisca-2 (9),6-diene (peak 3), (E)-β-caryophyllene (peak 4), and α-humulene (peak 5), β-elemene (peak 6), 4,5-diepi-isoishwarane (peak 7), 4,5-diepi-aristolochene (peak 8), eremophilene (peak 9), α-selinene (peak 10), and germacrene A (peak 11).

Plant-derived compounds can persist through insect digestion and be recovered in frass with limited metabolic degradation in insects [[40], [41], [42]]. To determine whether sesquiterpenes produced by *RlMTPSL3*+*RlMTPSL4*+*HMGR* after ingestion could contribute to growth suppression in herbivores, we examined frass from beet armyworm larvae fed on agroinfiltrated leaves. Headspace SPME-GC/MS analysis of frass collected from the beet armyworm larvae fed on *RlMTPSL3 + RlMTPSL4 + HMGR*-expressing *N. benthamiana* leaves revealed 11 sesquiterpene products (Fig. 6A). These sesquiterpene products previously detected in agroinfiltrated leaves, including asterisca-1,6-diene, the major product of *RlMTPSL3*, as well as the production of two sesquiterpenes, β-elemene and 4,5-diepi-isoishwarane, the characteristic products of RlMTPSL4 (Fig. 6A). Additional minor peaks matched other *RlMTPSL3*-and *RlMTPSL4*-derived sesquiterpenes based on retention indices and mass spectral profiles. In contrast, no such compounds were detected in frass from the larvae fed on emtpy vector control plant (Fig. 6B).

### 3.6 Emission of sesquiterpenes from intact leaves and their ecological function

Volatile sesquiterpenes synthesized *in planta* can be released into the headspace and function in ecological signaling [43,44]. Having established that *RlMTPSL3* and *RlMTPSL4* encode active sesquiterpene synthases [34] and that co-expression with *HMGR* enhances sesquiterpene yield (Fig. 3), we next examined whether these sesquiterpenes are emitted from intact leaves. Both *RlMTPSL3* and *RlMTPSL4*-derived sesquiterpenes including asterisca-1,6-diene, β-elemene and 4,5-diepi-isoishwarane along with their minor products were detected in the headspace of *N. benthamiana* leaves, demonstrating that engineered plants emit these compounds into the environment (Fig. 6C). These results demonstrate that engineered leaves not only accumulate sesquiterpenes internally but also emit them as volatiles, making them available for mediating ecological interactions.

Volatile terpenes are known to mediate insect behavioral responses, including repellence, feeding deterrence, and oviposition avoidance [45,46]. To evaluate whether these volatiles influence insect behavior, dual-choice bioassays were conducted with third-instar beet armyworm larvae. Across three independently aggregated biological groups (each comprising three technical trials), larvae consistently preferred control leaves over terpene-emitting leaves (Fig. 6D). Specifically, 57-67% of larvae selected control leaves, whereas only 20–22% selected terpene-emitting leaves, with 13-30% remaining in the neutral (“no choice”) zone. A one-tailed binomial test comparing engineered versus control selections within each biological group revealed that larvae chose engineered leaves significantly less frequently than control leaves (H_0_: p = 0.5; H_1_: p (engineered) < p (control)), indicating a robust avoidance response. Larvae that did not make a choice were excluded from statistical inference, consistent with standard two-choice assay practice.

### 3.7 Sesquiterpenes from *RlMTPSL3* and *RlMTPSL4* inhibit growth of the tomato wilt pathogen *Fusarium oxysporum*

**Fig. 7:**
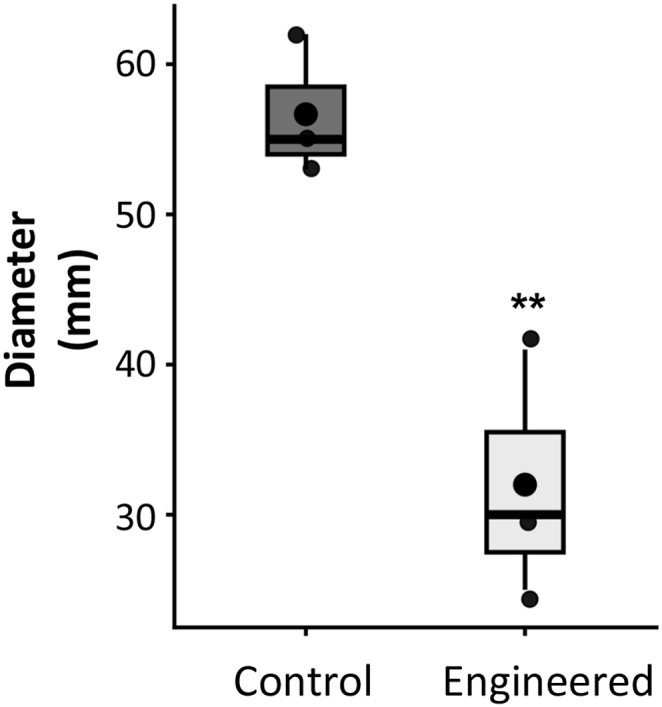
**Fungal growth bioassay using ethyl acetate extracts from agroinfiltrated *N*. *benthamiana* leaves**. Four-week-old *N. benthamiana* leaves were agroinfiltrated with *RlMTPSL3* + *RlMTPSL4* + *HMGR* or with an empty vector + *HMGR* as a control. Three days post-infiltration, leaf disks were excised, and insoluble metabolites were extracted with ethyl acetate. For each treatment, 400 μl of extract was evenly spread onto potato dextrose agar plates, after which a 5 mm cube of *Fusarium oxysporum* mycelium was placed in the center. Plates were incubated for 7 days before measuring fungal growth diameter. Data were analyzed using Student's *t*-test (*P*< 0.05). Asterisks indicate significance levels (∗*P* < 0.01*, P* < 0.05).

Plant-derived phytochemicals are well documented to inhibit fungal pathogens by disrupting membrane integrity and suppressing hyphal growth [12,47]. To determine whether terpenes produced by *RlMTPSL3* and *RlMTPSL4* also inhibit plant pathogens, we tested ethyl acetate extracts from agroinfiltrated *N. benthamiana* leaves against the tomato wilt pathogen *Fusarium oxysporum* f. sp. *lycopersici*. Fungal growth was markedly reduced in the presence of extracts from leaves co-expressing *RlMTPSL3*, *RlMTPSL4*, and *HMGR* compared with control treatments (Fig. 7; student's t-test, *P* < 0.05). Colony diameters were approximately 50% smaller than those of the empty-vector control, indicating substantial inhibition of mycelial expansion. These results indicate that *RlMTPSL3*-and *RlMTPSL4*-derived sesquiterpenes identified earlier as anti-herbivore compounds, also possess antifungal activity.

## 4 Discussion

In this study, we successfully engineered terpene metabolism in the model flowering plant *N. benthamiana* to produce new sesquiterpenes and demonstrated that these compounds can markedly influence plant-biotic interactions. These findings highlight a promising strategy for enhancing crop resistance to natural enemies. Our overarching goal was to expand the chemical diversity of terpenoids in flowering plants. Although terpenoid biosynthesis is ubiquitous across land plants and collectively generates an enormous array of structures [48], individual species produce only a small fraction of this diversity. Metabolic engineering of terpenoid pathways therefore offers a powerful means to artificially broaden terpenoid profiles, effectively serving as an accelerated form of chemical evolution.

Non-seed plants, particularly bryophytes, produce a remarkable diversity of terpenoids, many of which appear absent from seed plants [17]. In these early-diverging lineages, numerous terpenoids are synthesized by microbial-type terpene synthase-like (*MTPSL*) genes [17]. Although *MTPSL* genes have deep evolutionary roots within land plants, they are notably absent from seed plants [17], making them compelling candidates for introducing new terpenoid biosynthetic capacities into modern crops through metabolic engineering. In this study, we selected two *MTPSL* genes, *RlMTPSL3* and *RlMTPSL4*, from the liverwort *Radula lindenbergiana*, primarily because their major products represent previously unreported terpenoids [34], Fig. 1). Engineering these *MTPSL* genes into flowering plants was anticipated to be straightforward, as the biosynthetic pathway leading to their substrate, farnesyl diphosphate (FPP), is highly conserved across land plants [18]. The successful production of the *in vitro* products of RlMTPSL3 and RlMTPSL4 in tobacco leaves (Fig. 1, Fig. 2) confirms that these enzymes are functional in a heterologous seed-plant system and validates the feasibility of this metabolic engineering strategy.

Insect feeding on *N. benthamiana* leaves expressing *RlMTPSL3*+*RlMTPSL4*+*HMGR* led to a pronounced reduction in larval growth, suggesting that the newly synthesized terpenes imposed a strong physiological stress on the herbivore. This pattern reflects partial tolerance rather than full resistance, as larvae-maintained survival but with impaired performance [49]. Previous studies have shown that terpenes impair insect growth by deterring feeding, inhibiting digestive enzymes such as α-amylase and protease, and disrupting ecdysteroid-mediated molting and development in *Spodoptera* and other lepidopteran species [50]. Consistent with these mechanisms, two sesquiterpenes (alantolactone and isoalantolactone) from *Inula racemosa* reduce growth in *S. litura* by suppressing food consumption and lowering the efficiency of converting ingested and digested food into biomass [51]. Recent work further shows that sesquiterpenes disrupt larval growth by suppressing esterase and glutathione S-transferase activity in midgut of *S*. *litura* [52]. Other terpenoids show comparable potency: the sesquiterpene polyol ester angulatin A from *Celastrus angulatus* suppresses feeding and exhibits insecticidal activity in *Helicoverpa armigera* [53], while sesqui- and diterpenoids from *Porella chilensis* including pinguisanes, fusicoccanes, and an aromadendrane cause strong larval growth suppression and midgut epithelial disruption in *S. frugiperda* [54]. Comparable effects are seen in coleopterans, where the limonoid khayasin from *Neobeguea mahafalensis* interferes with nutrient uptake and digestion in *Batocera longissima* [55]. In this study, the strong inhibitory effects likely reflect a lack of evolutionary adaptation in the herbivore's detoxification system to the new sesquiterpenes produced in *N. benthamiana*. Such interactions exemplify how plants can expand their chemical repertoire to gain a transient advantage in the co-evolutionary arms race, until insect populations evolve metabolic or behavioral countermeasures to overcome these new defenses.

Beyond direct physiological inhibition, the new sesquiterpenes have altered the gut microbiome, possibly impairing detoxification capacity. In *Spodoptera frugiperda*, host plants rich in alkaloids, flavonoids, and phenolics significantly reshaped gut bacterial communities, enriching *Enterococcus*, *Enterobacter*, and *Acinetobacter*, while reducing overall diversity and metabolic pathways linked to terpenoid and xenobiotic metabolism [[56], [57], [58]]. Such dysbiosis reflects selective pressure from plant metabolites that suppress detoxifying symbionts and constrain microbial metabolism. A similar mechanism may have occurred in beet armyworm, where microbiome suppression or compositional shifts induced by the engineered sesquiterpenes could have weakened detoxification enzyme activity, contributing to the accumulation and excretion of unmetabolized compounds.

Metabolite profiling of larval frass revealed recovery of intact sesquiterpene ions, indicating minimal metabolic modification or conjugation. This suggests exclusion and rapid elimination rather than enzymatic degradation. Comparable patterns have been reported in other lepidopteran systems. Recent metabolomic profiling of *Laothoe populi* frass revealed that different classes of plant metabolites exhibit distinct fates during digestion: salicinoids were extensively cleaved and conjugated, whereas flavonoids were predominantly excreted unchanged, reflecting limited enzymatic modification or absorption [59]. Similarly, *S. littoralis* larvae feeding on *Origanum vulgare* retained only about half of the ingested terpenoids while excreting the remainder in frass [40]. In contrast, *S. litura* metabolized the simpler monoterpene β-myrcene to myrcene-3 (10)-glycol and myrcene-1,2-glycol through oxidative reactions independent of gut microbes [60]. The absence of comparable metabolites in our study suggests that *RlMTPSL3*+*RlMTPSL4*+*HMGR*-derived sesquiterpenes were too structurally complex or lipophilic to be recognized by insect oxidases. These results indicate a limited evolutionary capacity of *Spodoptera* detoxification systems to process chemically new sesquiterpenes, resulting in their exclusion and excretion in unmodified form.

In addition to post-ingestive effects, the newly produced sesquiterpenes functioned as potent olfactory repellents. Such pre-ingestive avoidance is a key adaptive behavior allowing herbivores to detect and evade toxic or metabolically costly compounds. Volatile-mediated deterrence has been reported in *Helicoverpa zea* and *S. exigua*, where sesquiterpene carboxylic acids from wild tomato (*Lycopersicon hirsutum* LA1777) reduced larval feeding and survival at concentrations above 2 mg/g leaf tissue [61]. Similar olfactory responses to terpene-rich volatiles have been observed in *Choristoneura occidentalis* and *Plutella xylostella*, which preferentially feed on tissues with reduced terpene or saponin levels [62,63]. This volatile-mediated avoidance complements the physiological inhibition observed in feeding assays, forming a two-layered defensive strategy repellence through perception and metabolic inhibition through ingestion. The strong repellence to *RlMTPSL3/4*-derived volatiles likely reflects a lack of prior evolutionary exposure in beet armyworm, precluding desensitization or habituation.

Beyond insect defense, terpenes are widely recognized for their antimicrobial properties [64]. For instance, capsidiol in *Nicotiana attenuata* suppresses *Alternaria alternata* infection [65], oryzalexins in rice provide resistance to *Magnaporthe oryzae* [66], and maize zealexins and kauralexins inhibit *Fusarium* and other fungal pathogens [67]. In our study, extracts containing *RlMTPSL3* and *RlMTPSL4*-derived compounds inhibited *F. oxysporum* growth, indicating dual defensive functionality. Thus, the engineered pathway may simultaneously reinforce both anti-herbivore and antimicrobial defense, enhancing overall plant resilience.

This study demonstrates a rational metabolic engineering strategy inspired by pathway evolution for expanding the chemical diversity of flowering plants by harnessing the biosynthetic potential of non-seed plant enzymes. With respect to target selection, *MTPSL* genes from the liverwort *R. lindenbergiana* were strategically chosen because their products represent chemically novel sesquiterpenes absent from flowering plants. With respect to pathway optimization, co-expression of HMGR, the rate-limiting enzyme of the MVA pathway, was designed to boost precursor flux and maximize sesquiterpene yields. With respect to functional validation, the ecological and defensive roles of the engineered metabolites were systematically evaluated across multiple biotic interactions, including herbivore growth suppression, gut microbiome disruption, volatile-mediated repellence, and antifungal activity. This work illustrates how the chemical diversity encoded in early-diverging plant lineages can be deliberately transferred into modern crop systems, offering an evolution-guided approach to engineering durable, multi-layered plant defenses against both insect herbivores and fungal pathogens.

## Author contributions

FC, CNS, SCL, and AO conceived the research. SUM performed the experiments, analyzed the data, and generated the figures. XC and BAO performed experiments and analyzed data. SUM and FC wrote the draft manuscript, BAO, AO, SCL and CNS reviewed and edited the manuscript. All authors approved the final version of the manuscript.

## Declaration of competing interest

The authors declare that they have no known competing financial interests or personal relationships that could have appeared to influence the work reported in this paper.

## Data Availability

The raw microbiome sequencing data generated in this study have been deposited in the NCBI Sequence Read Archive (SRA) under BioProject ID PRJNA1447733.
