# Supplementary material for: Microbial-type terpene synthases enable enhanced insect and fungal resistance in engineered plants
Source: Biodes Res. 2026 Apr 20;8(2):100087. doi: 10.1016/j.bidere.2026.100087 (PMC13137197; doi:10.1016/j.bidere.2026.100087)
Supplement: Multimedia component 1 [file mmc1.pdf]

**CsVMV promoter** (5’-3’)

CCAGAAGGTAATTATCCAAGATGTAGCATCAAGAATCCAATGTTTACGGGAAAAACTATGGAAGTATTATGTAAGCTCAGCAAGAAG
CAGATCAATATGCGGCACATATGCAACCTATGTTCAAAAATGAAGAATGTACAGATACAAGATCCTATACTGCCAGAATACGAAGAA
GAATACGTAGAAATTGAAAAAGAAGAACCAGGCGAAGAAAAGAATCTTGATGACGTAAGCACTGACGACAACAATGAAAAGAAG
AAGATAAGGTCGGTGATTGTGAAAGAGACATAGAGGACACATGTAAGGTGGAAAATGTAAGGGCGGAAAGTAACCTTATCACAAA
GGAATCTTATCCCCCACTACTTATCCTTTTATATTTTTCCGTGTCATTTTTGCCCTTGAGTTTTCCTATATAAGGAACCAAGTTCGGCAT
TTGTGAAAACAAGAAAAAATTTGGTGTAAGCTATTTTCTTTGAAGTACTGAGGATACAACTTCAGAGAAATTTGTAAGTTTGT

***ocs* terminator (5’-3’)**

CTGCTTTAATGAGATATGCGAGAAGCCTATGATCGCATGATATTTGCTTTCAATTCTGTTGTGCACGTTGTAAAAAACCTGAGCATGT
GTAGCTCAGATCCTTACCGCCGGTTTCGGTTCATTCTAATGAATATATCACCCGTTACTATCGTATTTTTATGAATAATATTCTCCGTTCA
ATTTACTGATTGTACCCTACTACTTATATGTACAATATTAAAATGAAAACAATATATTGTGCTGAATAGGTTTATAGCGACATCTATGATA
GAGCGCCACAATAACAAACAATTGCGTTTTATTATTACAAATCCAATTTTAAAAAAAGCGGCAGAACCGGTCAAACCTAAAAGACT
GATTACATAAATCTTATTCAAATTTCAAAAGTGCCCCAGGGGCTAGTATCTACGACACACCGAGCGGCGAACTAATAACGCTCACTG
AAGGGAACTCCGGTTCCCCGCCGGCGCGCATGGGTGAGATTCCTTGAAGTTGAGTATTGGCCGTCCGCTCTACCGAAAGTTACGGG
CACCATTCAACCCGGTCCAGCACGGCGGCCGGGTAACCGACTTGCTGCCCCGAGAATTATGCAGCATTTTTTTGGTGTATGTGGGCC
CCAAATGAAGTGCAGGTCAAACCTTGACAGTGACGACAAATCGTTGGGCGGGTCCAGGGCGAATTTTGCGACAACATGTCGAGGC
TCAGCAG

***RlMTPSL3* (domesticated for Golden Gate cloning removing *Bsa*I and *Bpi*I restriction sites)**

ATGGACGAAGGTATTTCACCACAAGATCTCGATCGCAGGATGGGGTCCATATCTGTGGCATATTTAGGCCAACAACAGATGTCCGAG
GAGGAGATCCGGAGGATAAAGTCCATGAGTGTGCCGCAATCGAAGTCTCCGTTTCCCGCAAAGACGAGCAAGCAAGAGAAGGAG
GCTGTGGCATACCTTGGCCAGAAGCAGATGTCCGAGGAGGAGATTCAAAGGATAAAAGCCATCAATCTGCCACAAGTGAAGTCTCC
GTATCCAGCAAAAACAAACAAGCACGCGAAGGAAGCATTGGCCGAGGCCCACACGTGGGCCCGCCAGCCCCCCCTGTGCAAAATG
TATAGGAGTCAGAAGGTGATGGATGCTCATACCGGCGCTGACTTCGACTTATGCGGGCTGGCGATGCGCGTGTATGAAGATGTGGAG
GAGAAACCAGCGGCCTTTTCTGGAAAGCTCATTCTCTGGACTTTTGCACTCGACGATTACATCGACGGCGGCGCCACCTTCGATGA
CCCTTCCAAGACGGCCGCGTTGCTTTGGGAGCTCAGCGCCATTATCATGTGGTCGTTTCCGGACCATCAATATCTCTGTCAGAACTTT
GCCAATGTTGTAAGCAGCGGGGATGCCACTCAACAGGATGCGGCTCTTGCTTGGATGAATCGCACGCTTGCCGACGCCAAACGCAA
TACGGGCACATTTTACGATACCTCTGCCGCGGATTGCTCGCCATTCTCCAAAGCACTAGGGGAGCTGTGGGCCACAGTCGCAGCGTC
GTCACCTCCAGAGTTTCTACTCCGGTTTGGCATTTCAATTCAGCGCTACGTCCTCAGCAATTTGACAGAGGTCATTAGTCGCAATTGC
AAAACCATATTGCCCCTCGCCGAGTACATCGAAGTGCGAAGGCGGTCCGTCGCCATGGAAGTATTTATGGTGATAGTTGAGTTTCTA
AACAATATATATCTGCCCGATGAAGTGTTCTTTACGCCAGGAATGCAGAGGATCATTACGGCAGCTAATGATATCGTTGCATGGCTCA
ACGATATCTGTTCTTTCAAGAAGGAGATATTACAGGGAGATCTGTGCAACTTGGTGGGAGTAATTAGCAACGAGCTAAATTGTACAT
TTGAAGAGGCTGCAGAAAGAGCATTCCTTATGAGCATGACCAGGATTGCAGACCTTGATAAAGATATAAGCGATTTGAGAAGAATTA
CTCCACCAGAGCACCGGACCGGGGTCGAAATGTACATTCAAGCCAGCACAAATTGGGCTTGGCGTAGCTACGAATGGTATTGCAAT
AGCAAACGTTATAATTTCGATGTGTGA

***RlMTPSL4***

ATGCCTACTACCGATTTCCGGCGACGAGAGAGAAAGTTCGTGCAAATGGGCACCGAAGTGGACCGACCCGCGCCTGTTCTTGACCA
CAGTCACGACAGTATCGCAAATCTAAAACCATTTGATCGGCAGCAAGAGCTGCGAATGTACAAGCCACCCCCTTTCATTCCTCCGTA
CCCCGTTAAACAGAATCCCAACTATTCGAAGGGAAGGTATTCGAACTGGGCTCGGAAATGGCTTCTAAACCTGAAGGTGGACGAGA
TATTCAGCACCAAAATGTTTGAGATGCTGAACCGCATGGACATTCCTTATTTTGCGAACGTTTTGCAGCCTTTGTGTGATGAGTGGG
GACTTCAGTGGTCGTGTAAGGTTTATTTCATGCTTTGGATAACGGACGACTTGGTGGACTCGACCAAAATCGGGAAATCTGCCTCTG
ATGTTTTGTCCCTATTTCTAGACTATCATTTGGTGATGATGTGGACGTTTCCGGACGACCCTGTTTTGCATAGGGAGCTCCCTAATTTT
CTCAGTGTTTTGGCGTCCCAAACCGAGCGCGAGCAAAAGCTGGCACATTTTGAGGAGATTTTGGTCCAAGCTAGGACTCGACCTGG
GACAATATACGAAGGGAATCTGTCAATTGCCTGTGAAGTATTCCGAGAGCTATTCGCTGAAGCATATGCAAGGTCGTCTCGCGAGTC
AGTTCTCCAGTTTGCACATTGCTGTCAACACTGGCTATTGGGAGATCTTCTGGAATCCCAATATCGAGAAGCGAAAGGAATGCCTGC
ATCAATTGAAGAATTGATACCAATCAGAAAGCGAGCCTGCGCAGTGTTCGTCGCATTGGCAAGCACAGATATTTCCTGTGGTCTGGC
TACTCCAACTGAGTACTACAATTCAAAGCCGATGAAGGAAATGCTTGATGCCTGTTGCGATTTCACGGCCTGGCACAATGATGTATG
GTCCTTCAAAAAGGAAATCATACAGGACAAGGAACCGTTTAGTCTTGTGGTAGCCATTAGTGTACATCGTAAATTGCCGTACCAGGA
GGCAGCTGAGGTTCTCACCAAGATGATGAGCGATAGGCTGAAGGACATGGACCGCGCAGCTACCGACTTGGAGCGTATTACGCCTC
CAGAACTGGCACGAAATTTTCAAATCTACGTGAGTGCCTGCCAGACTATGGCTTCTGGCACCCACGATTGGCATACCAAAACCGTG
CGCTATGATGTATAG

**Figure S1.** Sequences of the promoter, terminator and transgenes. The CsVMV promoter is from Cassava Vein Mosaic Virus, the ocs terminator is from *Agrobacterium tumefaciens*. Two transgenes *RlMTPSL3* and *RlMTPSL4* are from liverwort *Radula lindenbergiana*.

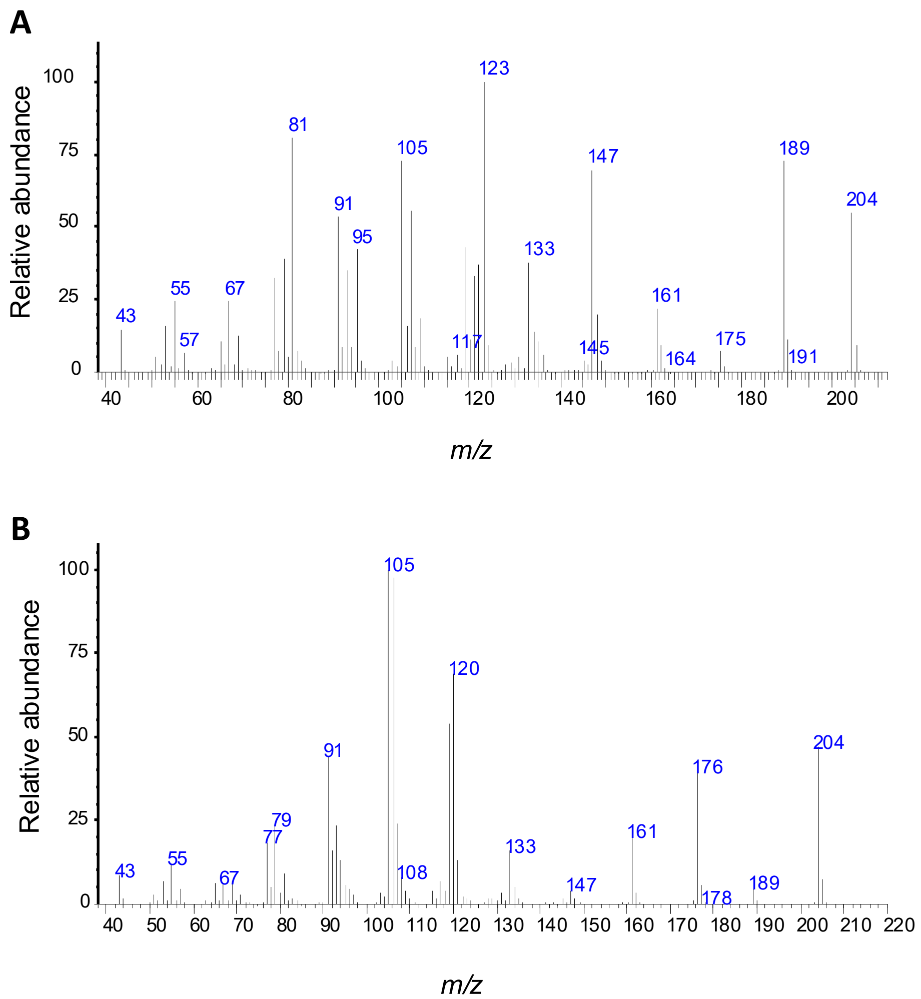


**Figure S2. Mass spectra of major sesquiterpene products of RlMTPSL enzymes.** (A) Mass spectrum of the major product of RlMTPSL3, asterisca-1,6-diene. (B) Mass spectrum of the major product formed by RlMTPSL4, 4,5-diepi-isoishwarane.
